# Application of network teaching in nursing undergraduate education during the coronavirus disease 2019 epidemic

**DOI:** 10.1186/s12909-022-03318-6

**Published:** 2022-04-01

**Authors:** Xiuwen Chen, Shiqing Liu, Zirong Tao, Yangyang Ou, Yao Xiao

**Affiliations:** 1 Teaching and Research Section of Clinical Nursing, Xiangya Hospital, Central South University, Changsha, China; 2 Department of Respiratory Medicine, Xiangya Hospital, Central South University, Changsha, China; 3 Branch of National Clinical Research Center for Respiratory Disease, Xiangya Hospital, Central South University, Changsha, China; 4 Department of General Surgery, Xiangya Hospital, Central South University, Changsha, China; 5 Department of Academic Affairs, Xiangya Hospital, Central South University, Changsha, China; 6 International Joint Research Center of Minimally Invasive Endoscopic Technology Equipment & Standards, Xiangya Hospital, Central South University, Changsha, China; 7 National Clinical Research Center for Geriatric Disorders, Xiangya Hospital, Central South University, Changsha, China

**Keywords:** Coronavirus disease 2019, Network teaching, Undergraduate, Nursing education

## Abstract

**Background and objectives:**

Coronavirus disease 2019 (COVID-19) is currently raging all over the world. COVID-19 can be transmitted among humans. To control the spread of the epidemic and meet the learning needs of students, Chinese universities have used a variety of multimedia forms to conduct online teaching. However, the influence of different teaching methods on the learning effects of nursing students has not been conclusive, and few studies have directly compared the differences in the effects of different teaching methods. Therefore, it is necessary to evaluate the impact of different teaching methods on students’ learning effects.

**Methods:**

A total of 119 participants from the graduating class of 2022 performed 4 validated classes (fundamental nursing, medical nursing, surgical nursing, and paediatric nursing) through network teaching. A total of 126 participants from the graduating class of 2021 used the traditional teaching method and were enrolled as a control group. All participants completed a questionnaire, which was designed by the school for teaching evaluation, and took a test after the courses.

**Results:**

A total of 245 nursing undergraduates were ultimately enrolled. The analysis of the academic performance and the student evaluations from the four courses showed no significant difference between the network teaching group and the traditional teaching group for nursing undergraduates (all *P* > 0.05).

**Conclusion:**

Through the convenience of network teaching, teachers and students can choose the time and place of both their teaching and learning independently. Moreover, such use effectively prevents the spread of the epidemic. After the epidemic concludes, the continued use of the network teaching method can help improve teaching efficiency by being combined with the traditional teaching method.

**Supplementary Information:**

The online version contains supplementary material available at 10.1186/s12909-022-03318-6.

## Introduction

At present, coronavirus disease 2019 (COVID-19) continues to rage across the world [1, 2]. Based on the findings of current epidemiological investigations and studies, respiratory droplets and direct contact transmissions are the main transmission routes of COVID-19, and a risk of transmission exists in relatively closed environments in which people are exposed to high concentrations of aerosols for a long time. People are generally susceptible to COVID-19 [3, 4]; therefore, isolation is the most common and effective means to prevent infection. The traditional teaching method refers to students and teachers gathering in the classroom for face-to-face teaching; obviously, there is a higher risk for the spread of the epidemic when using this method. Emerging technologies offer innovative ways to deliver online education and revolutionize how students think, learn, and construct knowledge [5]. The use of network learning and the use of educational technology are rapidly becoming popular in higher education [6]. To avoid the spread of the epidemic on campuses, network teaching has been more widely carried out in China. Due to the convenience of the internet, both teachers and students can choose their own times and places for teaching and learning independently. Online teacher-student interactions not only enable students to master the learning content efficiently but also effectively prevent the spread of the epidemic. However, as online education is a brand-new teaching mode, the influence of different teaching methods on learning and teaching effects has not yet been made conclusive. Research by Luo Shuhong and colleagues showed that online learning is particularly attractive to nurses because they often have to take into account the combined responsibilities of school, family, and work [6]. Debra Hampton also found that teacher self-efficacy can be facilitated through faculty development and increased experience with teaching online [7]. Online delivery has been identified as a solution for expanding program capacity and increasing access to nursing education [8]. Conversely, Micki’s data showed that content containing complex cognitive concepts, experiential learning in regard to relational practice, and psychomotor skill mastery are better suited for traditional classroom delivery [9]. Other researchers have also found that only 29.1% of chief academic officers in the U.S. believe that their faculty has accepted the “value and legitimacy of online education” [10]. Although the above studies have begun to focus on online education, in terms of research content, most of the studies were based on the evaluation of teachers’ or students’ acceptance of online education or their satisfaction, and the research methods used were mainly qualitative research and cross-sectional surveys. Few studies have evaluated the actual performance of students’ online education, and even fewer studies have directly compared the teaching effects of different teaching methods. Therefore, it is necessary to evaluate the impact of network teaching and traditional teaching on students’ learning outcomes.

Undergraduate education is the initial stage of medical education. Laying a solid foundation through undergraduate education is conducive to stimulating students’ interest in learning and influencing their future career choices. Even during the epidemic situation of COVID-19, attention should be given to undergraduate education; internet teaching can help make up for the suspension of classes caused by the epidemic. To ensure the high-efficiency learning quality of students and complete the undergraduate training plan, our university began to apply network teaching based on a visual teaching platform for the teaching activities of nursing undergraduates on February 17, 2020. The purpose of the research in this article is to analyse and compare academic performance and student evaluations in network teaching and traditional teaching for nursing students.

## Materials and methods

### Study population

**Fig. 1:**
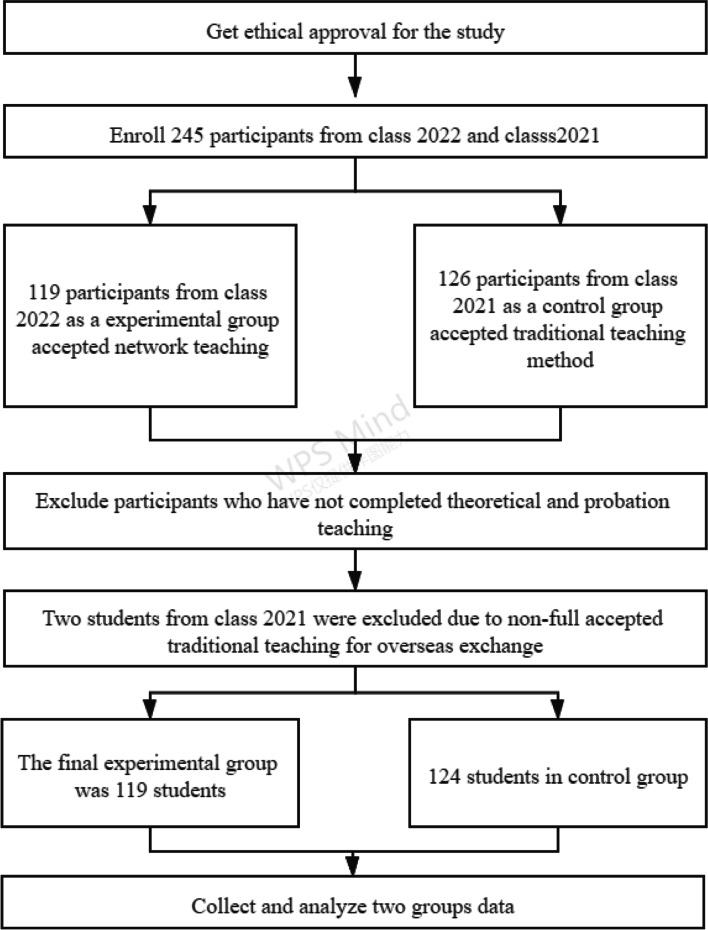
Flow chart of the study design. Nursing undergraduates from the graduating class of 2022 who used the network teaching method were enrolled. For the control group, we also enrolled participants from the graduating class of 2021 who used the traditional teaching method (students and teachers gathered in the classroom for face-to-face teaching)

A convenience sample of nursing students from the graduating class of 2021 and the graduating class of 2022 was selected as the object of this study. The inclusion criteria were as follows: (a) nursing undergraduates and (B) those with full-time undergraduate enrolment. The exclusion criteria were (a) dropouts or those with an academic suspension, (b) failure to participate in theoretical teaching or probation teaching, and (c) those with psychological or mental illness. Nursing undergraduates from the graduating class of 2022 were enrolled in courses using the network teaching method. For the control group, we enrolled participants from the graduating class of 2021 in courses using the traditional teaching method (i.e., students and teachers gather in the classroom for face-to-face teaching) (Fig. 1). Students from both the graduating class of 2021 and the graduating class of 2022 also enrolled in four test classes.

### Study design

This study design was a historical controlled trial. Members of the graduating class of 2021 had experienced the traditional teaching method during the previous year, while the graduating class of 2022 used the network teaching method to learn the same course information. All the participants completed questionnaires and took tests after the courses. The number of class hours was the same for two groups. Each semester, the four courses of fundamental nursing, medical nursing, surgical nursing, and paediatric nursing required 76, 72, 72, and 58 lessons, respectively. Moreover, the precourse knowledge of the two groups of students was examined before the experiment. A knowledge questionnaire was designed by us, mainly to evaluate the knowledge that students had learned in the previous semester. The knowledge questionnaire consisted of 10 multiple-choice questions; one point was awarded for each correct answer, for a total of score of 10. The computed Cronbach’s alpha based on the collected data was 0.832, which indicates acceptable reliability.

To ensure the smooth implementation of network teaching, all teachers received 2 days of unified training before engaging in online education. The training mainly focused on the operation and use of visual teaching platforms, including how to cultivate internet thinking; how to design and organize online teaching; how to use the platform to upload, download and collect teaching resources; how to use interactive functions to carry out teacher-student interactions; how to post learning tasks; and how to use mobile phones to connect to platforms.

### Hypotheses

The primary hypothesis was that the academic performance of the two types of courses would show no significant difference between the network teaching group and the traditional teaching group for nursing undergraduates. The secondary hypothesis was that no significant difference would be found in the student course evaluations between the two groups.

### Theoretical teaching

The teachers made the teaching contents available according to a weekly calendar, which could be in the form of a video recording. A class hour was generally 20-30 min. At the beginning of the course, a personal introduction was given to show the teacher’s style to the students. At the end of the course, a two-dimensional code was required to be displayed for students to evaluate the course (Table S1). The reliability and validity of the student evaluation instrument tool was confirmed; the Cronbach’s alpha value of Table S1 was 0.921, and the item content validity index (I-CVI) ranged from 0.915-1.000. In-class thinking questions were arranged so that students could independently study and complete these in-class thinking questions within the corresponding period of time. In-class teachers answered questions and helped to organize students to interact with each other within the specified period of time. A secretary who was in charge of course construction collected subsequent relevant questions and feedback from students and summarized and submitted them to the lecturing teacher for unified reply. Students’ attendance and test results were recorded as reference indicators for final assessment. In short, four steps, including in-class tests and intensive questions and answers (Q&A), were completed after class feedback was received and the weekly performances were recorded to track each student’s learning path.

### Probation teaching

TronClass platform preclass preview: this preview consisted of a systematic review (video) of relevant knowledge points, case-related learning materials (documents), cases for discussion (3 cases), cases for inquiry simulation (1-2 cases), extension cases (1-2 cases).

Preclass group discussion among students (no teacher participation): each group (15-17 people) was divided into three subgroups for group discussion, and one case was discussed in each group. Each student explained and described the case (in the form of oral presentation, PPT or video).

In-class discussion (teacher-led participation): each group (15-17 people) was organized by a teacher in order to participate in online discussion. Analytical thinking and discussions about the result of the case were elaborated in subgroups. Both students and teachers could ask questions or make additions, and the teachers made a brief summary of the discussion.

Online inquiry simulation: a teacher played the role of a patient, a student played the role of a doctor, and simulation teaching was integrated into online teaching.

Summative comment and feedback: the teacher made summative comments, gave targeted feedback and assigned related homework after class (Table S2). The Cronbach’s alpha value of Table S2 was 0.937, and the I-CVI ranged from 0.865-1.000.

Both theoretical teaching and probation teaching are indispensable parts of the four courses. The absence of either of these styles will directly affect the quality of students’ education. Theoretical teaching focuses on basic theoretical knowledge and the basis of study and brings correct and reasonable theory and a basis of study to the phenomena in practice. Probation teaching is uses theory to practice and pay attention to clinical skills training.

### Quality control

From the aspects of teaching design, communication and interaction, teaching evaluation, learning support and feedback for improvement, this paper puts forward important observation points regarding online course teaching quality. Under the guidance of the researchers, the online teaching quality evaluation form was developed as the evaluation basis for supervision and peer listening (Table S3). The reliability and validity of this tool was also confirmed; the Cronbach’s alpha value of Table S3 was 0.892, and the I-CVI ranged from 0.890 to 1.000. All students had to accept both theoretical teaching and probationary teaching. The students who did not complete the process were excluded from the study. Each course of the two groups was taught by corresponding teachers in the same teaching group. In addition, to reduce the bias stemming from differences in test questions, we controlled the two groups of students such that they had same type of test questions, and the content of the tests was all within the requirements of the syllabi to ensure that the difficulty of the questions was consistent. All the questions come from the same question bank, and the average scores of the students who had received traditional education in recent years were also close together.

### Statistical analysis

All statistical analyses were carried out using SPSS 19.0 (SPSS Inc., Chicago, IL). The data values are presented as the mean ± standard deviation (SD). Differences in the mean values between the two groups were analysed by two-tailed Student’s t tests. A *P* value < 0.05 was considered statistically significant.

## Results

### Study population

In total, 245 nursing undergraduates were enrolled. Two students from the graduating class of 2021 were excluded due to their incomplete acceptance of traditional teaching for overseas exchange. A total of 119 participants from four test classes were included as the network teaching group, and 126 participants from four test classes were included as the traditional teaching group. There were no statistically significant differences in age (20.00 ± 0.748 vs. 19.97 ± 0.867 years, *p* = 0.76), sex (males: 11.76% vs. 11.90%, *p* = 0.973), or birthplace (town: 52.94% vs. 53.97%, *p* = 0.872) between the two groups (Table 1).

**Table 1:** Participants’ demographic characteristics

Characteristics	Network teaching group	Traditional teaching group	*P*
Group size	119	126	
Age, years (mean, SD)	20.00 ± 0.748	19.97 ± 0.867	0.76
Gender (%)			0.973
Male	14 (11.76)	15 (11.90)	
Female	105 (88.24)	111 (88.10)	
Birthplace (%)			0.872
Town	63 (52.94)	68 (53.97)	
Village	56 (47.06)	58 (46.03)	
Precourse knowledge questionnaire scores (mean, SD)	8.07 ± 1.550	7.87 ± 1.955	0.392

### Academic performance

The analysis of the academic performances within the four courses showed no significant difference between the network teaching group and the traditional teaching group for nursing undergraduates (all *P >* 0.05) (Table 2). However, the surgical nursing course indicated that online education was borderline inferior to in-person education according to the American Statistical Association’s guidelines since the *p* value was 0.074.

**Table 2:** Academic performance comparison

	Network teaching group	Traditional teaching group	t	*P*
Fundamental nursing	82.27 ± 8.105	81.53 ± 7.823	0.724	0.470
Medical Nursing	82.98 ± 5.526	82.31 ± 7.513	0.803	0.423
Surgical nursing	75.51 ± 7.216	77.18 ± 7.329	1.796	0.074
Paediatric nursing	85.15 ± 4.661	84.49 ± 7.088	0.865	0.388

### Evaluation of courses

The analysis of the student evaluations of the four courses showed no significant difference between the network teaching group and the traditional teaching group for nursing undergraduates (all *P >* 0.05) (Table 3).

**Table 3:** Student evaluation comparison

	Network teaching group	Traditional teaching group	t	*P*
Fundamental nursing	92.36 ± 7.291	93.27 ± 8.512	0.896	0.371
Medical Nursing	93.26 ± 6.342	94.04 ± 8.632	0.802	0.423
Surgical nursing	95.78 ± 9.672	94.63 ± 8.561	0.987	0.325
Paediatric nursing	89.03 ± 5.987	90.34 ± 6.091	1.697	0.091

## Discussion

The current analysis found no differences between the network teaching group and the traditional teaching group regarding academic performance and the evaluation of courses for nursing undergraduates. At present, the rapid and constant evolution of large online courses, such as massive open online courses (MOOCs), has attracted the attention of the educational community and achieved widespread popularity among many universities [6, 11]. Chan MM et al. have shown that using online videos as learning resources is very useful for students in disciplines such as health and medicine, as evidenced by increased student participation within the courses; in addition, medical students expressed their desire to continue learning with network courses [11]. Network teaching relies on a network platform for online teaching; this mainly includes two forms, namely, live online instruction and video recording. Compared with the traditional method, network teaching more easily arouses students’ interest and supports playback, which is conducive for students to review and consolidate information after class [12]. Students can repeatedly review the knowledge points or process steps that they did not understand initially. In addition, this study showed that compared with other courses, the academic performance of students in the surgical nursing course for online education was slightly worse than that for traditional education. One reason for such an outcome may be related to the strong practicality of surgical nursing. The surgical nursing course requires a flexible combination of theoretical knowledge and clinical cases to further confirm and strengthen book knowledge [13]. However, due to the small sample size of this study, it was difficult to draw a conclusion; thus, further research is needed in the future.

### Advantages of the network teaching mode in undergraduate nursing education

#### Sufficient network teaching resources

In addition to the courses recorded by teachers, we can also make full use of the existing MOOC resources to seamlessly connect with the existing teaching programs. The courses are constructed in a centralized way, and the service orientation is accurate and efficient. Teachers can modify and improve their course repeatedly to strive for perfection during the process of rerecording, and course resources are deposited as precious supplies for future teaching. As a learning management platform, the visualization platform integrates the characteristic teaching modules such as open classes, resource databases, micro-course recordings, learning circles, teaching evaluations and learning analyses, which have obvious advantages in teaching process management [14, 15]. The teaching resources are updated quickly. The COVID-19 topic, as a knowledge point of emerging infectious diseases, can be added to the existing curriculum as a chapter. The teachers who are fighting in the anti-epidemic frontline can quickly turn their first-hand clinical data into high-quality teaching cases.

#### Standardized and efficient process management

Attention is given to teaching process management to ensure learning effects. Four steps, namely, in-class tests, intensive Q&A, after class feedback and recording weekly performances, were completed to track each student’s learning path. To ensure the effect of group teaching, a teacher-student ratio of approximately 1:15 was strictly implemented, and sufficient discussion time was ensured. The simulation teaching method was used to increase students’ intuitive understanding of the cases. Attention was given to learning both before and after class, and students were encouraged to think actively step by step; mistakes were allowed, and it was emphasized that students should create a summary of the course. The weekly internship calendar was adjusted; the courses that require actual on-site observation and practice should be arranged at the end of the term. A variety of online learning materials should be provided in the early stage of the program, which should be be smoothly connected with the courses after students return to school to ensure teaching quality.

#### Flexible teaching time and place

Network teaching creates network classrooms. Some medical teachers have rushed to the anti-epidemic frontline because of their work needs; however, they can record their courses anytime and anywhere after finishing their clinical work. Generally, the length of a course is set as 20-30 min. The key points and difficult points are highlighted. Students can learn independently according to their own situation and replay the video of corresponding content repeatedly to learn difficult points. Meanwhile, the online interaction between teachers and students increases the students’ level of interest in learning and the degree of student participation.

### Deficiency and prospect of network teaching mode in medical undergraduate education

An internship course is not suitable for online teaching because the internship course requires student participation. Also, because only an account name and password are required to log in, the system would not be able to identify whether the user is the student himself/herself or not; thus, the situation in which a student who attends a class falsifies the attendance of another student who is absent or of some students who failed an exam cannot be avoided. Furthermore, poor internet signals in mountainous areas and remote areas bring difficulties to network teaching. At the same time, the platform is not perfect, and the Caton phenomenon existed during the application process; it is difficult for students with poor subjective initiative and weak self-control ability to effectively acquire knowledge through network teaching. Moreover, the lack of interaction is a problem in web-based teaching [16]. These insufficiencies of network teaching reminded us that to adapt to the rapid development of information technology, medical colleges and universities must consider the COVID-19 epidemic as an opportunity to quickly update their educational concepts, cultivate teachers’ online thinking, innovate online teaching models, and make full use of the network platform to provide better teaching and services.

At present, network teaching is in the gradual improvement stage. In view of the above shortcomings, the visual teaching platform has a video feedback function, which can identify the user’s dynamic situation and put an end to situations in which a student who attends a class falsifies the attendance of another student who is absent or of some students who failed an exam. The visual teaching platform integrates the online courses of medical colleges and universities, collects the results of student evaluations, and increases the results of lecturing teachers or peer expert evaluations to sift out excellent courses for teaching use. During such periods as the COVID-19 epidemic, using such a special teaching method will give students more time to think and exercise their active learning ability.

## Limitations

First, due to the sudden COVID-19 epidemic outbreak, the Chinese Ministry of Education proposed “continuing teaching and learning regardless of suspending classes” to provide “available courses and teachers” for students, which limited this study to only a historical controlled trial. Since the grouping of traditional teaching and network teaching was not random, the authenticity of the results is reduced by the influence of selection bias, and the strength of the conclusion could thus be weakened. Second, the sample of available teachers was small, and our study did not assess teachers’ feelings about online education; thus, we cannot provide comprehensive information. We plan to conduct multicentre and qualitative research in this field in future work. Third, there is rapid advancement in the technology in terms of the software and hardware used in the learning tools. These technology changes are also reflected in studies related to network teaching. As a result, students’ perceptions of network education change dynamically. We should also conduct longitudinal studies in future research to determine how students’ performances in network teaching change.

## Supplementary Information

**Additional file 1: Table S1.** Student evaluation on theory courses.

**Additional file 2: Table S2.** Student evaluation on internship course.

**Additional file 3: Table S3.** Supervisor evaluation form.

## Data Availability

All data generated or analysed during this study are included in this published article.
