# Supplementary material for: Application of network teaching in nursing undergraduate education during the coronavirus disease 2019 epidemic
Source: BMC Med Educ. 2022 Apr 1;22:231. doi: 10.1186/s12909-022-03318-6 (PMC8972715; doi:10.1186/s12909-022-03318-6)
Supplement: Supplementary file 1 — Additional file 1: Table S1. Student evaluation on theory courses. [file 12909_2022_3318_MOESM1_ESM.docx]

Table S1 Student evaluation on theory courses

| Observation indicators | [Evaluation grade](javascript:;) | | | |
| --- | --- | --- | --- | --- |
|  | A(10) | B(8) | C(6) | D(4) |
| 1. The teacher pays attention to the cultivation of ideals and beliefs and socialist core values |  |  |  |  |
| 1. The teacher has strict requirements for education contents such as medical humanistic care and professional spirit |  |  |  |  |
| 1. The teacher is animated, has dignified appearance and can timely release teaching resources |  |  |  |  |
| 1. Teaching design is scientific, and preparation of teaching materials is sufficient |  |  |  |  |
| 1. Teaching objectives are clear, contents are rich and practical, and key and difficult points are highlighted |  |  |  |  |
| 1. The teach combines theory with practice, pays attention to ability training, and timely introduces subject frontier knowledge |  |  |  |  |
| 1. The teach gives lessons in mandarin, with concise, accurate and vivid expressions |  |  |  |  |
| 1. The teaching contents are matched with other forms of reference learning materials such as literature |  |  |  |  |
| 1. The teacher is able to stimulate and promote thinking and active learning among students |  |  |  |  |
| 1. The teacher can always help the students to maintain a high level of focus on learning |  |  |  |  |
