# Supplementary material for: Application of network teaching in nursing undergraduate education during the coronavirus disease 2019 epidemic
Source: BMC Med Educ. 2022 Apr 1;22:231. doi: 10.1186/s12909-022-03318-6 (PMC8972715; doi:10.1186/s12909-022-03318-6)
Supplement: Supplementary file 2 — Additional file 2: Table S2. Student evaluation on internship course. [file 12909_2022_3318_MOESM2_ESM.docx]

Table S2 Student evaluation on internship course

| Observation indicators | [Evaluation grade](javascript:;) | | | |
| --- | --- | --- | --- | --- |
|  | A(10) | B(8) | C(6) | D(4) |
| 1. The teacher pays attention to combining the content to carry out the education of entrepreneur spirit, values, morality or humanistic quality |  |  |  |  |
| 2. The teacher cares for students, communicates with students and answer their questions actively |  |  |  |  |
| 3. The teacher issues cases for group discussion, clarifies the basic theories and knowledge points involved in the discussion, which meet the teaching requirements |  |  |  |  |
| 4. The teacher conducts a systematic review of case-related knowledge points |  |  |  |  |
| 5. The teacher plays a leading role (takes control of the class and topics, and corrects errors in the use of theory, knowledge, and technology in the discussion, and makes comments on discussion) |  |  |  |  |
| 6. The teacher actively enlightens students' scientific thinking, cultivates students' innovative consciousness, pays attention to two-way communication between teachers and students, and provides rich content and sufficient time for group discussion. |  |  |  |  |
| 7. The teacher can guide group students to report and discuss scientific, knowledgeable and exploratory contents, which meet the subject requirements. |  |  |  |  |
| 8. The teacher can guide students to deliver keynote speeches(express clearly and display clear content) |  |  |  |  |
| 9. The teacher can induce students to actively participate in group discussion or debate, and classroom atmosphere is active |  |  |  |  |
| 10. Self evaluation: the students have a high participation degree, can ask questions actively, gain a lot, and flexibly use basic theoretical knowledge and technical skills |  |  |  |  |
