# Supplementary material for: Application of network teaching in nursing undergraduate education during the coronavirus disease 2019 epidemic
Source: BMC Med Educ. 2022 Apr 1;22:231. doi: 10.1186/s12909-022-03318-6 (PMC8972715; doi:10.1186/s12909-022-03318-6)
Supplement: Supplementary file 3 — Additional file 3: Table S3. Supervisor evaluation form. [file 12909_2022_3318_MOESM3_ESM.docx]

Table S3 Supervisor evaluation form

| Indicators | Observation points | Weight | [Evaluation grade](javascript:;) | | | |  |
| --- | --- | --- | --- | --- | --- | --- | --- |
|  |  |  | A(10) | B(8) | C(6) | D(4) | |
| Teaching preparation | 1. Before class, the teacher put forward the course learning requirements, the composition and proportion of the usual grades, the course evaluation method and the grading method to the students. | 0.1 |  |  |  |  | |
|  | 2. The curriculum structure is reasonable and rich learning resources are pushed. | 0.1 |  |  |  |  | |
| Teaching process | 3. Teaching design reflects the characteristics of online teaching, which is conducive to mobilizing students' thinking and learning enthusiasm (selection of time and content). | 0.1 |  |  |  |  | |
|  | 4. Teaching reflects "two natures and one degree (high level, innovative and challenge degree)", focuses on ability cultivation, and timely introduces cutting-edge knowledge of the subject. | 0.1 |  |  |  |  | |
|  | 5. Teaching resources are closely related to teaching aims (including teaching syllabus, PPT, lecture notes, video, audio, etc.). | 0.1 |  |  |  |  | |
|  | 6. The teacher can communicate and interact with students, discuss and answer questions in various ways. | 0.1 |  |  |  |  | |
|  | 7. The visual teaching platform is used effectively, the tests, voting questionnaires and homework are arranged reasonably; pre-class test and post-class homework are closely related to teaching contents. | 0.1 |  |  |  |  | |
| Teaching effect | 8. Students generally use the learning resources pushed by teachers effectively. | 0.15 |  |  |  |  | |
|  | 9. Students actively participate in teaching activities, the interaction between teachers and students is good, and students basically master the teaching content. | 0.15 |  |  |  |  | |
